# Incidence, patterns and severity of reported unintentional injuries in Pakistan for persons five years and older: results of the National Health Survey of Pakistan 1990–94

**DOI:** 10.1186/1471-2458-7-152

**Published:** 2007-07-10

**Authors:** Zafar Fatmi, Wilbur C Hadden, Junaid A Razzak, Huma I Qureshi, Adnan A Hyder, Gregory Pappas

**Affiliations:** 1 Department of Community Health Sciences, Aga Khan University, Karachi, Pakistan; 2 Health and Human Services, Center for Disease Control, Atlanta, USA; 3 Department of Emergency Medicine, Aga Khan University, Karachi, Pakistan; 4 Pakistan Medical Research Council, Islamabad, Pakistan; 5 Johns Hopkins Bloomberg School of Public Health, Baltimore, USA

## Abstract

**Background:**

National level estimates of injuries are not readily available for developing countries. This study estimated the annual incidence, patterns and severity of unintentional injuries among persons over five years of age in Pakistan.

**Methods:**

National Health Survey of Pakistan (NHSP 1990–94) is a nationally representative survey of the household. Through a two-stage stratified design, 18, 315 persons over 5 years of age were interviewed to estimate the overall annual incidence, patterns and severity of unintentional injuries for males and females in urban and rural areas over the preceding one year. Weighted estimates were computed adjusting for complex survey design using *surveyfreq *and *surveylogistic *option of SAS 9.1 software.

**Results:**

The overall annual incidence of all unintentional injuries was 45.9 (CI: 39.3–52.5) per 1000 per year; 59.2 (CI: 49.2–69.2) and 33.2 (CI: 27.0–39.4) per 1000 per year among males and females over five years of age, respectively. An estimated 6.16 million unintentional injuries occur in Pakistan annually among persons over five years of age. Urban and rural injuries were 55.9 (95% CI: 48.1–63.7) and 41.2 (95% CI: 32.2–50.0) per 1000 per year, respectively. The annual incidence of injuries due to falls were 22.2 (95% CI: 18.0–26.4), poisoning 3.3 (95%CI: 0.5–6.1) and burn was 1.5 (95%CI: 0.9–2.1) per 1000 per year. The majority of injuries occurred at home 19.2 (95%CI: 16.0–22.4) or on the roads 17.0 (95%CI: 13.8–20.2). Road traffic/street, school and urban injuries were more likely to result in handicap.

**Conclusion:**

There is high burden of unintentional injuries among persons over five years of age in Pakistan. These results are useful to plan further studies and prioritizing prevention programs on injuries nationally and other developing countries with similar situation.

## Background

Unintentional injuries are the main cause of injury deaths worldwide [1-3]. More than two-thirds of injuries occur in developing countries [4,5]. Deaths from injuries are projected to increase from 5.1 million to 8.4 million (9.2% of all global deaths) and injuries are estimated to be the third leading cause of disability adjusted life years (DALYs) by the year 2020 [6,7].

Despite the high burden developing countries have just begun to systematically address injury control in policies and programs [8]. This lack of programmatic and policy response to injury is in part due to lack of population-based and national estimates of injuries in developing countries [9]. Hospital-based statistics and police records are the common sources of information for injuries in developing countries [10-16]. However, these sources underestimate the burden because they systematically under-report and capture only the most severe cases on injuries [1].

Few small scale studies have estimated the injuries in Pakistan [17,18], and also a nationally representative survey, the National Injury Survey of Pakistan (NISP), was conducted in 1997 [19]. The NISP provided estimates of overall injury, but publications from it focused on road traffic injuries only. Therefore, Pakistan lacks population based estimates of the burden of common injuries such as falls, burns and farm/field related injuries.

In this paper we use the National Health Survey of Pakistan (NHSP 1990–94) to provide an epidemiological profile of unintentional injuries in the country. The Government of Pakistan is planning to conduct a second National Health Survey and also plans to launch a national policy and program to address injuries in Pakistan. We present the annual incidence estimates of overall injuries in Pakistan using NHSP, which is the most recent health examination survey in Pakistan to guide future research and policy action regarding injury in the country. Additionally, the survey provides annual incidence estimates for types (falls, poisoning, burns, crushing/breaking/bruises related to road traffic) and places (homes, roads, farms and schools) of injuries in Pakistan, and information on the proportion of injuries by urban and rural location and gender. This paper also examines the levels of recovery from injuries as proxy for severity of injuries, and explores relationships between these variables.

## Methods

The National Health Survey of Pakistan (NHSP) was conducted between 1990 and 1994 by the Pakistan Medical Research Council (PMRC) and the Federal Bureau of Statistics (FBS), with technical assistance from the National Center of Health Statistics (NCHS) of the US Centers for Disease Control (CDC). An ethical review committee (oversight committee) reviewed and approved the survey following the guidelines of Helsinki Declaration.

The survey sample had a two-stage stratified design [20]. The urban and rural areas (according to 1981 population census) of each of the four provinces of Pakistan (Punjab, Sindh, NWFP, Balochistan) were taken as strata. Based on the master sampling frame of the FBS, enumeration blocks (EBs) of approximately 200–250 households in the urban strata and villages [or *dehs *or *mozas*] in the rural strata were taken as primary sampling units (PSUs). There were 80 PSUs selected for the survey. From each unit 30 households were sampled using a systematic sampling technique by taking a random start and a sampling interval. All residents of the 2,400 households were included in the study. Overall, 18,315 persons were interviewed after taking the informed consent from the individuals (or proxy respondents) and assent for younger individuals. Of these, 15,078 were over 5 years of age. Data collection began in February 1990 and was completed in August 1994.

Operationally, the data was collected in two major parts: a household interview (from the head of household) and health examination and interview of the every individual from the selected household. From the individual over five years of age a series of questions were asked about unintentional injuries that occurred over the past 12 months. Proxy respondents (usually mothers) were questioned for persons less than 12 years of age. Unintentional injuries were defined as any injury event which was not considered deliberate and for which medical treatment was sought. While the older term 'accident' was used in the survey instrument, we report them as unintentional injuries in this paper. The study instrument was developed in English and then translated into Urdu, Punjabi, Pushto, Sindhi, and Balochi [20]. The word accident was translated in Urdu and other languages as *"Hadsa" *which means unintentional injury event.

Also questions were asked about the place, type and recovery status from injuries. Injury types included all events related to any cause – poisoning, falls, burns and crushing/breaking/bruises related to road traffic. The place of injury included home, roads, workplace, fields/farms, and schools. Injury victims were asked if at the time of the interview they had 'fully recovered', 'partially recovered', or 'recovered with handicap.'

Quality control for the survey included visits to the field by expert consultants, duplicate interview by field supervisors and retraining exercises.

Analysis was done using SAS 9.1 software. Weighted analysis was performed. The *suveyfreq *and *surveylogistic *options were used to adjust for design effect of the survey and get valid estimates [21]. Injury incidence rates were calculated based on 1000 persons and disaggregated analysis was performed by age, gender, educational status and location of residence. Injury incidence for types (falls, poisoning, burns, crushing/breaking/bruises related to road traffic) and places (home, road, farms and school) were also calculated. Uni-variate analysis was performed exploring relationships between injuries and variables of age, gender, educational status and location of residence. Multivariate analysis was performed to get adjusted odds ratios, controlling for other variables for injuries and taking age, gender, and educational status and location of residence together into consideration. The analysis was restricted to persons 5 years and older since the injury questions included were very different for younger ages in the data collection strategy. It was assumed that most of the injuries for younger than 5 would occur at home. Data missing on variables were < 5% and were excluded from analysis and *n *for individual variables has been reported in the tables, wherever it was necessary. Confidence intervals were reported at 95% level throughout this study. Places and types of unintentional injuries were also disaggregated by gender, and urban and rural location to observe the proportion of various categories of injuries. Distributions of recovery from injuries were analyzed to understand the severity of injuries.

## Results

The overall annual incidence of unintentional injuries was estimated at 45.9 (CI: 39.3–52.5) per 1000 per year for Pakistanis over the age of five years (Table 1). Applied to the current population [22] this rate implies a total burden of 6.16 million injuries per year for persons over five years of age in Pakistan. The incidence of injuries increases with age and was highest among the > 45 years age group. Overall, injuries were more common among males than females, and there were more injuries in urban than in rural areas. Injuries were more common among those who had some literacy compared to those who were not literate. Respondents reported falls more often than other types of injuries, and they reported that injury occurred most often in the home or as a result of road traffic.

**Table 1:** National annual incidence of reported injuries and injury types for persons over five years of age by socio-demographic and urban/rural status in Pakistan (NHSP, 1990–94)

**Variables**	**Frequency**	**Incidence per 1000 per year**	**95% CI**
**Overall injuries **(n = 15078)	678	45.9	(39.3 – 52.5)
**Type of injuries**			
Fall	339	22.3	(18.0–26.5)
Poisoning	42	3.3	(0.5–6.1)
Burn	22	1.5	(0.9–2.1)
Other*	272	18.8	(15.2–22.2)
**Place of injuries**			
Home	284	19.2	(15.5 – 22.9)
Road traffic/Street	252	17.0	(13.8 – 20.2)
Workplace/Fields/Farm	85	6.1	(4.3 – 7.7)
School	27	1.6	(0.8 – 2.2)
Other	31	2.0	(1.1 – 2.9)
**Age categories (years)**			
5–15 (n = 5960)	252	42.6	(36.4 – 48.8)
16–30 (n = 4142)	189	46.2	(36.2 – 56.2)
31–45 (n = 2366)	108	46.9	(36.7 – 57.1)
> 45 (n = 2571)	129	52.7	(40.3 – 65.1)
**Gender**			
Female (n = 7676)	248	33.2	(27.0–39.4)
Male (n = 7345)	430	59.2	(49.2–69.2)
**Education**			
Literate (n = 4372)	254	60.2	(50.2–70.2)
Not literate (n = 6761)	260	39.7	(30.7–48.7)
**Location of residence**			
Urban (n = 5437)	296	55.9	(48.1–63.7)
Rural (n = 9584)	382	41.2	(32.2–50.2)

*Source: *NHSP 1990–94.

*Injuries related to body part crushing, breaking and bruising.

Table 2 presents the odds ratios for age, gender, education, and urban/rural location independently and adjusted odds from a multi-variate model. Older age was associated with injury after controlling for the other variables, though of marginal significance. Overall injuries were 87% more common among males than among females, controlling for the other variables. The risk of injuries among person with some literacy compared to no literacy and in urban compared to rural areas was not significantly different.

**Table 2:** Odds ratio and adjusted odds ratios of reported injuries for persons over five years of age by socio-demographic and urban/rural status in Pakistan (NHSP, 1990–94)

**Variables**	**OR**		**95% CI**	**AOR**		**95% CI**
**Age categories**						
5–15 years	1.0			1.0		
16–30 years	1.1		0.9–1.3	1.1		0.9–1.4
31–45 years	1.1		0.9–1.4	1.2		0.9–1.6
> 45 years	1.3		1.0–1.6	1.4	*	1.0–1.9
**Sex**						
Female	1.0			1.0		
Male	1.8	*	1.5–2.3	1.9	*	1.5–2.4
**Education**						
Not literate	1.0			1.0		
Literate	1.5	*	1.2–2.1	1.3		0.9–1.7
**Location of residence**						
Rural	1.0			1.0		
Urban	1.4	*	1.1–1.8	1.2		0.9–1.6

*Source: *NHSP 1990–94. *p ≤ 0.05

The distribution of reported places and types of injuries is presented in Table 3. Injuries occurring at home accounted for 42% and road traffic/street 38% of the total injuries. Fall was the most common type of injury. Table 3 also presents the distribution of reported places and types of injuries by gender. Home injuries accounted for the highest proportion of injuries among females, while road traffic injuries were the most common place of injuries among males. A high proportion of falls was reported among both males and females.

**Table 3:** Distribution of reported place and types of injury for persons over five years of age by gender and urban/rural residence in Pakistan (NHSP, 1990–94)

**Variables**	**Overall**	**Females (n = 7676)**	**Males (n = 7345)**		**Urban (n = 5458)**	**Rural (n = 9620)**	
	**Percent (SE)**						

**Place of injuries**							
Home	41.8 (2.6)	65.4 (2.8)	28.2 (3.6)	**	40.3 (2.7)	42.8 (4.0)	*
Road traffic/Street	37.6(2.7)	22.6 (2.9)	46.2 (3.8)		41.1 (2.8)	35.2 (4.1)	
Workplace/farm/fields	13.0(1.6)	6.9 (1.7)	16.5 (2.1)		8.1 (1.8)	16.3 (2.4)	
School	3.2(0.8)	3.8 (1.2)	2.9 (0.8)		5.7 (1.4)	1.5 (0.7)	
Others	4.4(0.9)	1.3 (0.8)	6.2 (1.3)		4.8 (1.1)	4.2 (1.3)	
**Type of injuries**							
Fall	48.6(2.8)	62.8 (2.8)	40.5 (3.6)	**	52.6 (4.5)	45.9 (3.7)	*
Poisoning	7.1(3.0)	3.2 (1.1)	9.4 (4.6)		1.9 (1.0)	10.6 (5.0)	
Burn	3.2(0.6)	5.1 (1.3)	2.1 (0.8)		3.6 (1.1)	3.0 (0.8)	
Other*	41.1(2.96)	28.9 (2.7)	48.0 (4.1)		41.9 (4.9)	40.5 (3.7)	

*Source: *NHSP 1990–94. **p ≤ 0.001 *p ≤ 0.05 ≥ 0.01

*Injuries related to body part crushing, breaking and bruising.

In both urban and rural areas the homes and road traffic were the most common locations of injuries and these were about equal in proportion (Table 3). In rural areas, in addition workplace/farm/fields related injuries were of greater proportion compared to urban areas. Falls were the most frequent type of injuries in both urban and rural areas, with poisoning contributing a greater proportion in rural areas than in urban areas.

To more fully understand the distributions of injury in the population we examined the recovery status associated with the injury, a proxy for the severity of the injury. Table 4 provides the recovery status of those reporting injury by age, gender, urban/rural status, types and places of injury. Those who reported 'full recovery' tended to be younger and male. Injury due to poisonings and burns, and injuries at home were more likely to be reported as 'fully recovered'. 'Handicaps' were more strongly associated with injuries that occurred on streets/roads, schools and injuries in urban areas. Paradoxically, injuries occurring in schools were also the group most likely to have 'recovered fully'. The type of injury most likely resulted in handicaps were those related to crushing/breaking/bruises related to road traffic.

**Table 4:** Distribution of recovery of injury for injured persons over five years of age by gender, urban/rural residence, place and type of injury in Pakistan (NHSP, 1990–94)

**Variables**	**Fully recovered**	**Under recovery**	**Recovered with handicap**	
	**Percent (SE)**			

**Age categories**				
5–15 years	75.3 (3.6)	18.5 (3.2)	6.2 (1.5)	**
16–30 years	66.0 (4.0)	27.2 (3.7)	6.8 (1.4)	
31–45 years	65.8 (4.9)	28.7 (5.0)	5.6 (2.1)	
> 45 years	52.6 (4.9)	41.5 (5.2)	5.8 (2.3)	
**Gender**				
Female	53.3 (4.0)	41.0 (4.7)	5.7 (1.5)	**
Male	74.2 (2.8)	19.3 (2.3)	6.5 (1.4)	
**Location of residence**				
Urban	67.3 (3.4)	23.6 (2.5)	9.1 (2.1)	*
Rural	66.2 (3.8)	29.5 (3.8)	4.3 (1.0)	
**Type of injuries**				
Fall	61.8 (3.4)	32.9 (3.3)	5.3 (1.0)	*
Poisoning	88.8 (7.2)	7.8 (5.7)	3.4 (3.1)	
Burn	77.2 (9.4)	18.1 (8.1)	4.7 (4.6)	
Other*	67.8 (3.5)	24.3 (2.9)	7.9 (2.1)	
**Place of injuries**				*
Home	68.9 (3.9)	26.6 (3.9)	4.5 (1.2)	
Road traffic/Street	63.5 (3.0)	27.4 (2.5)	9.1 (2.0)	
Workplace/Field/Farm	64.0 (6.0)	31.6 (6.0)	4.4 (2.2)	
School	74.2 (12.4)	16.1 (8.7)	9.7 (7.8)	
Other	76.8 (9.1)	23.2 (9.1)	0	

*Source: *NHSP 1990–94. **p ≤ 0.001 *p ≤ 0.05 ≥ 0.01

*Injuries related to body part crushing, breaking and bruising.

## Discussion

The National Health Survey of Pakistan (NHSP) was the first national survey to measure the annual incidence of unintentional injuries for persons over five years of age living in households in Pakistan. Unintentional injuries are the main cause of injury deaths and disability both in the developing and developed countries [1,2]. Because the NHSP collected information about the morbidity in a household population, mortality was not included in these estimates. Deaths due to unintentional injuries in Pakistan are estimated to be 0.68 per 1000 per year [19] and might be added to the annual incidence presented here for the living population. Also, only injuries which needed medical advice were counted and as such minor injuries were not included in the estimates, again underestimating the overall unintentional injuries. Because the information was collected for a recall of 12 months from individuals some injuries may have been forgotten, again leading to an underestimate of the true annual incidence. However minor injuries may more easily be forgotten than an injury requiring medical attention as has been reported by other researchers [23].

The estimates of unintended injuries can be compared to the National Injury Survey of Pakistan (1997) [19]. The sampling methods and definition used by the two surveys were similar. Both the NHSP and the NISP collected information on injuries that required medical advice or treatment. The estimates of overall incidence of unintended injuries of the two surveys were not different statistically: NISP reported 41 (CI: 39.2–43.8) injuries per 1000 per year and NHSP reports 45.9 (CI: 39.3–52.5) per 1000 per year [19]. NISP survey categorized the injuries into transport and non-transport related injuries only and did not report rates of other common injuries which occur at homes, schools and farm/field. Therefore, further pattern of injuries cannot be compared among the two surveys. Another community-based estimates in an urban squatter settlement of Karachi, Pakistan report an overall major injury events of 42 (CI: 41–43) per 1000 per year. Although this community-based survey was conducted on a small scale and not a nationally representative sample, highlights the importance of home injuries similar to NHSP [18]. It is also important to note that the three surveys were carried out in different time periods (NHSP-1990–94; community-based survey-1995; NISP-1997) which may point to the fact that the injuries rates may not have changed significantly during these periods in Pakistan.

The results of NHSP, however, suggest that the magnitude of injury is much greater than estimated by some other studies in Pakistan. These other studies, however, are small in size and are not population-based estimates. Some studies are hospital based or estimate injuries from emergency departments of hospitals and ambulance services or police records, therefore grossly undercount injuries. While some other studies have extrapolated findings from various sources [6,17,19,24,25]. Population-based estimates of injuries from developing countries are still uncommon and any reliable comparison cannot be made because of variable definitions used in the surveys. Although, available estimates suggest that Pakistan falls in the lower range of injury rates among the developing countries. A study in four rural communities in China estimated an incidence rate of 65.1 per 1000 per year [26]. On the other hand a study in Uganda estimated the total incidence of fatal and non-fatal injuries at 116 per 1000 per year. While in another study in Ghana overall incidence of minor and major injuries was 411 per 1000 person-years where more than one injury during the year was also counted [27,28].

Results from NHSP and NISP broadly agree. Age was not a significant factor in predicting injuries as was also reported by NISP [19]. Males were twice likely to have injuries than females in Pakistan. These finding are consistent with other studies from developing countries [1,2,19]. This clearly relates to the gender roles of males and females. Men are more likely to be involved in work outside homes and on the street/roads than women in Pakistan. It is estimated that only 3% of women were employed outside of the home in Karachi, the biggest city of Pakistan [30].

Injuries occurring in the home are common and falls were the most common type of injury occurring there. This finding agreed with a population based study in Karachi, in which fall was the most common type of injuries [18]. There should be further studies of in-home injuries to investigate their pattern and causes to develop preventive measures. There has been little emphasis on developing safety programs for in-home injuries in Pakistan and developing countries [31].

Although home injuries were the most common place of injury in Pakistan, road traffic injuries also occur at high levels and may be more serious in nature. This study suggests that road traffic injuries causes more handicap than do home injuries. The high prevalence and high severity of road/street injuries suggest that these should receive priority in research and policies and programs.

The recovery status associated with injuries provides us with some inferences about the severity of the events. The lower levels of full recovery among women and the high levels of handicaps resulting from injuries that occur in schools require further investigation. The chances of handicap following injury were double in the urban areas and it may reflect a lower case-fatality rate for accidents in towns and cities. While recovery status is not a direct measure for severity of injuries, these data presented to indicate patterns that can be useful in prioritizing future research and policy action, and to direct attention to the needs of subgroups in the population. Level of recovery depends on time since injury, therefore the category of 'under recovery' separates handicap from those who were undergoing recovery. The overall disabilities caused by injuries in Pakistan are much higher than reported in population based surveys in Uganda and Ghana [27,29].

The proportion of road traffic injuries in urban and rural areas were found equivalent, a finding supported by NISP [19]. Careful investigation needs to be carried out to look at the reasons for the high rate of road traffic injuries in rural areas. Furthermore, workplace/farm/fields injuries were one of the common causes of injuries in rural areas. Further studies should investigate the types of farm related injuries and their causes in rural areas.

It should be noted that NHSP data are now 12 years old. Pakistan has seen many changes during this time period. There has been a significant increase in traffic and urbanization [32], perhaps partly due to increasing numbers of automobiles recently made possible by consumer credit from banks in Pakistan [33]. The information presented in this paper focuses on persons over five years of age; pattern of injuries differs in younger population and it needs a different sort of investigation. It can also be assumed that most injuries among under-5 population occur at homes. Also, only morbidity due to injuries has been included in the estimates presented here. However, the value of community based and nationally representative data makes this analysis of critical importance to inform future research and decisions in Pakistan.

While interpreting the data it should also be kept in mind that one year recall period for the study lead to underestimation of injury events, however severe injuries (which need medical advice) are better recalled even after a period of one year (28). Also multiple injuries during the year in the same person were not recorded in this survey. It also underestimates the burden of injuries as it has been found that injuries may be clustered among particular individuals (34).

## Conclusion

The NHSP demonstrates that there is a high burden of unintentional injuries in Pakistan. Falls and road traffic are the two major categories of injuries and perhaps road traffic injuries causes more handicap and are a major challenge for the population. This survey should help researchers to conduct further focused studies on in-home, road traffic and farm related injuries nationally and internationally. This may also help policy makers in Pakistan to improve health systems for this significant burden to society. The Government of Pakistan is currently planning a national program to address unintentional injuries and also planning to conduct a second National Health Survey in Pakistan. These results would help in both these initiatives as a guide and a baseline estimates for injuries in Pakistan.

## Competing interests

The author(s) declare that they have no competing interests.

## Authors' contributions

GP and WH were technical consultants for the design and implementation of the National Health Survey of Pakistan. AH was a consultant to NHSP during analysis phase. ZF and GP conceived, designed, and wrote the first draft of the manuscript. ZF managed and analyzed the data, and wrote the complete draft of the manuscript. JR, AH and HQ gave technical input in writing the draft manuscript. WH supervised the statistical analysis. All authors reviewed, read and approved the final manuscript.

## Pre-publication history

The pre-publication history for this paper can be accessed here:


